# Atypical protein disulfide isomerases (PDI): Comparison of the molecular and catalytic properties of poplar PDI-A and PDI-M with PDI-L1A

**DOI:** 10.1371/journal.pone.0174753

**Published:** 2017-03-31

**Authors:** Benjamin Selles, Flavien Zannini, Jérémy Couturier, Jean-Pierre Jacquot, Nicolas Rouhier

**Affiliations:** UMR 1136 Interactions Arbres/Microorganismes, Université de Lorraine/ INRA, Faculté des Sciences et Technologies, Vandoeuvre-lès-Nancy, France; Universidade de Sao Paulo Instituto de Biociencias, BRAZIL

## Abstract

Protein disulfide isomerases are overwhelmingly multi-modular redox catalysts able to perform the formation, reduction or isomerisation of disulfide bonds. We present here the biochemical characterization of three different poplar PDI isoforms. PDI-A is characterized by a single catalytic Trx module, the so-called *a* domain, whereas PDI-L1a and PDI-M display an *a-b-b’-a*’ and *a°-a-b* organisation respectively. Their activities have been tested *in vitro* using purified recombinant proteins and a series of model substrates as insulin, NADPH thioredoxin reductase, NADP malate dehydrogenase (NADP-MDH), peroxiredoxins or RNase A. We demonstrated that PDI-A exhibited none of the usually reported activities, although the cysteines of the WCKHC active site signature are able to form a disulfide with a redox midpoint potential of -170 mV at pH 7.0. The fact that it is able to bind a [Fe_2_S_2_] cluster upon *Escherichia coli* expression and anaerobic purification might indicate that it does not have a function in dithiol-disulfide exchange reactions. The two other proteins were able to catalyze oxidation or reduction reactions, PDI-L1a being more efficient in most cases, except that it was unable to activate the non-physiological substrate NADP-MDH, in contrast to PDI-M. To further evaluate the contribution of the catalytic domains of PDI-M, the dicysteinic motifs have been independently mutated in each *a* domain. The results indicated that the two *a* domains seem interconnected and that the *a°* module preferentially catalyzed oxidation reactions whereas the *a* module catalyzed reduction reactions, in line with the respective redox potentials of -170 mV and -190 mV at pH 7.0. Overall, these *in vitro* results illustrate that the number and position of *a* and *b* domains influence the redox properties and substrate recognition (both electron donors and acceptors) of PDI which contributes to understand why this protein family expanded along evolution.

## Introduction

Oxidative protein folding is an essential process, occurring generally in oxidizing sub-cellular compartments, and which is required, in particular, for the maturation and assembly of newly synthesized secreted and membrane proteins. In the periplasm of prokaryotes, the proteins responsible for the formation and isomerisation of disulfide bonds belong to the Dsb (DiSulfide Bonds) protein family. This protein family is composed of several members referred to as DsbA to DsbL [1, 2]. The major actors, conserved in many bacteria, are the DsbA-DsbB couple which is involved in oxidation reactions, the DsbC-DsbD couple in isomerisation reactions, DsbE or CcmG in cytochrome c maturation and DsbG in the reduction of sulfenic acids [2–4]. In eukaryotes, the proteins responsible of the oxidative protein folding are essentially found in the endoplasmic reticulum (ER) and in the intermembrane mitochondrial space. In the ER, the proteins involved belong mostly to the endoplasmic reticulum oxidase (ERO) and protein disulfide isomerase (PDI) families [5]. In addition, proteins named quiescin sulfhydryl oxidases (QSOX) displaying oxidase activity *in vitro* are also present in this compartment and various physiological functions were proposed for QSOX [6–8]. In the mitochondrial intermembrane space, the oxidation of cysteines is ensured by the Mia40-Erv1 disulfide relay [9].

All PDI, as well as some Dsb members, belong to the thioredoxin (Trx) superfamily displaying a common structural fold named the Trx fold [10, 11]. All PDI possess at least one Trx domain referred to as *a* or *b* domain which consists of about 100 amino acids and adopts a Trx fold. The difference is that *a* modules exhibit a catalytic active site (most frequently WCGHC) whereas *b* modules do not possess the typical CxxC catalytic motifs but instead are thought to be important for substrate recognition [12, 13]. The isoforms usually differ by the number and the positions of *a* and *b* domains and by the active site signature of *a* domains. The classical PDI isoforms, which are present in all eukaryotic organisms, possess four modules with an *a*-*b*-*b’*-*a’* organisation. Several PDI isoforms possess a more limited number of domains as well as additional domains that have been evolutionary added and which likely confer specific functions to the isoforms possessing them [5].

PDI constitute a multigenic family composed of 5 genes in yeast and up to 20 genes in human [14, 15]. In photosynthetic organisms, PDI cluster into 9 classes (A, B, C, D, E, F, L, M and S), 3 are specific to algae (D, E, F), 1 is specific to land plants (A) and the 5 others are present in both phyla [5, 16–19]. Land plants possess around 10 members [5]. Some soybean PDI isoforms belonging to classes L, M and S play essential roles in seed and pollen maturation and development [17–22]. The plastidial form of a *Chlamydomonas reinhardtii* PDI-L is involved in the redox-dependent transcriptional regulation in the chloroplast [23]. A maize PDI-A protein is accumulated in response to an ER stress, but, in contrast to classical PDI, it might not reside in the ER or only transiently [16]. It was also recently proposed that the *Hordeum vulgare* PDI5.1 isoform, a PDI-A, is involved in the susceptibility toward various bymoviruses and particularly the barley yellow mosaic virus [24]. However, very little is known concerning the biochemical and physiological properties of many plant PDI.

Thus, the aim of this study was to compare the biochemical properties of three poplar PDI (PDI-A, PDI-L1a and PDI-M) exhibiting different domain organisations and belonging to three different classes, by examining in particular their *in vitro* reductase and oxidase/isomerase activities using both physiological and non-physiological substrates. For poplar PDI-M, a deeper analysis was performed using proteins with mutated active site cysteines in order to investigate the importance of each catalytic domain for the oxidation or reduction of disulfide bonds. The data indicated that, although the redox potentials of the two modules of PDI-M are very close, each Trx module preferentially performed specific reactions, the *a°* domain being rather competent for oxidation and the *a* domain for reduction.

## Material and methods

### Cloning, site-directed mutagenesis, production and purification of recombinant proteins

The sequences encoding poplar PDI-L1a (Potri.002G082100.1), PDI-M (Potri.014G160000.1), PDI-A (Potri.009G004500.2) were amplified from leaf cDNAs of *Populus trichocarpa x Populus deltoides* for PDI-L1a and *P*. *trichocarpa* for PDI-M and PDI-A, and cloned into pET3d for PDI-L1a or into pET-15b for PDI-M and PDI-A. In order to express proteins in their mature forms, the 21, 23 and 26 N-terminal amino acids corresponding to the putative ER targeting sequences in PDI-L1a, PDI-M and PDI-A respectively were removed. The primers used for cloning and site-directed mutagenesis (PDI-A K56G, PDI-M C36/C39S and C165/C168S) are listed in Table 1. Recombinant plasmids were used to transform the *Escherichia coli* BL21(DE3) pSBET strain. The culture conditions were as described in [25]. The purification of PDI-L1a consisted in a succession of ammonium sulfate precipitation, ACA44 gel filtration and DEAE Sephacel chromatography as described [25]. The purification of His-tagged PDI-M and PDI-A variants was performed by affinity chromatography on IMAC columns (Sigma Aldrich) from the soluble fraction obtained after a 30 min centrifugation (27,000 x g) of cells lysed by sonication. The washing buffer was 30 mM Tris–HCl pH 8.0, 300 mM NaCl, 10 mM imidazole and the elution buffer was 30 mM Tris–HCl pH 8.0, 300 mM NaCl, 250 mM imidazole. All proteins were finally dialyzed against a 30 mM Tris-HCl pH 8.0, 1 mM EDTA, 200 mM NaCl buffer by ultrafiltration on YM10 membranes and concentrated. The same procedure was applied for the anaerobic purification of PDI-A in the glove box except that imidazole was removed by desalting on a G25 column and the protein was concentrated using 500 μL centricon centrifugal filters omitting EDTA in the buffer. Protein concentrations were determined using molar extinction coefficients at 280 nm of 39,100 M^−1^ cm^−1^ for PDI-L1a, 55,265 M^−1^ cm^−1^ for PDI-M, 55,140 M^−1^ cm^−1^ for PDI-M C36/C39S and PDI-M C165/C168S, 21,220 M^−1^ cm^−1^ for PDI-A and PDI-A K56G.

**Table 1:** Oligonucleotides used in this study for cloning and site-directed mutagenesis experiments.

name	sequences
PDI-L1a for	5’CCCCCCATGGCTGAGGATGAATCAAAGGAGTAC 3’
PDI-L1a rev	5’CCCCGGATCCTCAAAGTTCATCTTTAGCTGT 3’
PDI-M for	5’CCCCCCCCCATATGCTATATGGGCCTTCATCTCCT 3’
PDI-M rev	5’CCCCGGATCCTTATAACTCATCCTTGCTTCC 3’
PDI-M C36/39S for	5’GCACCATGGTCTGGGCACTCTAAAGCTCTC 3’
PDI-M C36/39S rev	5’GAGAGCTTTAGAGTGCCCAGACCATGGTGC3’
PDI-M C165/168S for	5’GCACCTTGGTCGGGTCACTCTAAGAAACTGGCT3’
PDI-M C165/168S rev	5’AGCCAGTTTCTTAGAGTGACCCGACCAAGGTGC3’
PDI-A for	5’CCCCCCCCCATATGGTTATAACCCTAACTCCT 3’
PDI-A rev	5’CCCCGGATCCTCACAAATCTTTATCATAGCC 3’
PDI-A K56G for	5’TGTGTTCCCTGGTGTGGGCATTGTAAGAATTTG 3’
PDI-A K56G rev	5’CAAATTCTTACAATGCCCACACCAGGGAACACA 3’

Restriction sites are underlined.

### Oligomerization state determination of PDI-A

The oligomerization state of PtPDI-A was analyzed using an ÄKTA Purifier system equipped with a Sephadex75 10–300 column (GE Healthcare). 100 μg of fresh anaerobically-purified proteins reduced in the presence of a 10-fold DTT excess at room temperature was loaded on the column at a flow rate 0.5 ml min–1, and detection was recorded at 280 and 420 nm. The columns were calibrated using the 29–700 kDa molecular weight standards (Sigma).

### Determination of the redox midpoint potential

Oxidation-reduction titrations using the fluorescence of adducts formed between the protein and monobromobimane (mBBr) were carried out at ambient temperature as described previously [26, 27]. The 500 μl reaction mixtures contained 100 μg protein in 100 mM HEPES-NaOH buffer pH 7.0, together with defined mixtures of oxidized and reduced glutathione to set the ambient potential (E_h_) with an overall concentration in glutathione of 2 mM. After 2 h incubation at 25°C, excess mBBr was added and incubated in the dark for 1 h. mBBr labelled proteins were precipitated with TCA 20% (v/v), incubated 15 min on ice and centrifuged 15 min at 13,000 rpm. Pellets were washed with 1 volume of 2% TCA by centrifugation (15 min, 13,000 rpm) and then resuspended in 300 μl Tris-HCl 1 M, SDS 2% during 30 min. Protein solutions were then diluted to a final volume of 2.2 ml and mBBr fluorescence emission was measured at 480 nm using a Cary Eclipse spectrofluorimeter after excitation at 350 nm. Fluorescence emission values were then plotted against Eh value for each sample with QtiPlot software and fitted with Boltzmann sigmoid preset parameters.

### RNase A oxidative refolding

RNase A reduction and denaturation was performed in 200 μl of 100 mM Tris-HCl pH 8.0 containing 6 M guanidine, 73 mM DTT and 7.3 mM native bovine RNase A (Sigma Aldrich), for 1 h at 37°C. Excess DTT was removed with a desalting column (Sephadex G25) equilibrated with 30 mM Tris-HCl pH 8.0, 1 mM EDTA and 1% acetic acid. The RNase A concentration was determined using a molar extinction coefficient at 280 nm of 9,800 M^-1^. cm^-1^. The effective full reduction of RNase A was confirmed with DTNB (5,5’-dithio-bis-2-nitrobenzoic acid, 13,600 M^-1^ cm^-1^) confirming the expected number of 8 thiols per RNase A molecule. RNase A refolding was assessed by incubating 50 μM of reduced and denatured RNase A for 15 min in a 300 μl mixture containing 30 mM Tris-HCl pH 8.0, 1 mM EDTA, 1 mM GSH, 0.2 mM GSSG with or without 3 μM PDI. For RNase A activity measurement, 50 μl of refolding mixture were added every 5 min to 450 μl of 30 mM Tris-HCl pH 8.0, 1 mM EDTA containing 2.5 mM cytidine 2',3'-cyclic monophosphate (cCMP). RNase A recovered activity was estimated by following the increase of absorbance at 296 nm resulting from the hydrolysis of cCMP to CMP [28].

### Insulin and 5,5′-Dithio-Bis-2-Nitrobenzoic Acid (DTNB) reduction

Insulin reduction was measured using 5 μM poplar Trx h1 or PDI as in [29]. The non-enzymatic reduction of insulin by DTT was used as a control. The ability of PDI to catalyze the reduction of DTNB in the presence of *Arabidopsis thaliana* NADPH-thioredoxin reductase B (AtNTRB) was measured at 25°C by monitoring the increase in absorbance at 412 nm caused by the release of thionitrobenzoate (TNB^−^). The reaction medium contained 30 mM Tris-HCl pH 8.0, 1 mM EDTA, 200 μM NADPH, 2 μM AtNTRB, 100 μM DTNB and 0.2 μM PDI.

### PDI-mediated recycling of 2-Cys Peroxiredoxin (Prx)

The PDI-dependent recycling of *A*. *thaliana* 2-Cys Prx was measured using a NADPH-coupled spectrophotometric method at 25°C as described previously [30]. The assays were carried out in a total volume of 500 μl containing 30 mM Tris-HCl pH 8.0, 1 mM EDTA, 200 μM NADPH, 1 μM AtNTRB, 5 μM At2-Cys Prx, 100 μM H_2_O_2_ and 2.5 μM PDI. The decrease in absorbance was followed at 340 nm. The peroxidase activity was determined after subtracting the spontaneous reduction rate observed in the absence of PDI.

### NADP-Malate Dehydrogenase (NADP-MDH) activation

The purification and activation test of NADP-MDH from *Sorghum bicolor* are as described in [31]. The activation mixture (200 μl) contained 500 μM DTT, 5 μM PDI or Trx h1 used as a control and 10 μg recombinant NADP-MDH in 100 mM Tris-HCl pH 8.0 buffer. It was incubated at 25°C for 15 min. Every 5 min, an aliquot of 20 μl was added to 480 μl of standard reaction mixture containing 100 mM Tris-HCl pH 8.0, 190 μM oxaloacetate, and 800 μM NADPH. Activities were measured by following the decrease in absorbance at 340 nm.

### Quantification of iron and sulfide contents

Protein concentrations of PDI-A have been estimated using the bicinchoninic acid (BCA) protein assay (Interchim) using bovine serum albumin as a standard. For iron measurements, known concentrations of PDI-A prepared in 130 μl are mixed with 90 μl of 1 M perchloric acid (PCA). After 15 min incubation at room temperature, the reaction mixtures are centrifuged at 10,000 x g for 5 min. Then, 144 μl of 3.15 mM bathophenantroline-disulfonic acid, 72 μl of 192 mM sodium ascorbate and 152 μl of 6.2 M ammonium acetate are sequentially added to 180 μl of the supernatant. The reaction mixtures are incubated 30 min at room temperature and centrifuged at 10,000 x g for 5 min. The absorbance ratio between 535 and 680 nm is determined for each sample including the standard curve (from 0 to 150 μM) made using a 500 μM ammonium iron(II) sulfate solution prepared by a 1/20 dilution from a 10 mM solution prepared in 1 N HCl.

For sulfide measurements, known concentrations of PDI-A prepared in 100 μl are mixed with 300 μl of 1% zinc acetate, immediately followed by the addition of 15 μl of NaOH 3N. The solution is vigorously mixed and incubated at room temperature for 10 min. Then, 75 μl of a 0.1% N,N-dimethyl-p-phenylenediamine (DMPD) solution prepared in HCl 5N are added, immediately followed by the addition of 16 μl of a 23 mM iron chloride solution prepared in HCl 1.2 N, and the mixture is vigorously mixed. The reaction mixture is then incubated for 30 min (standard curve) or 3h (samples) at 4°C. All samples are centrifuged 5 min at 10,000 x g and the sulfide concentration is determined by measuring the absorbance at 670 nm. The standard curve (0 to 100 μM) is prepared from a 200 μM Li_2_S solution prepared by a 1/100e dilution from a 20 mM stock solution prepared in 0.3 N NaOH.

## Results

### The three selected poplar PDI have particular sequence characteristics and domain organisations

**Fig 1:**
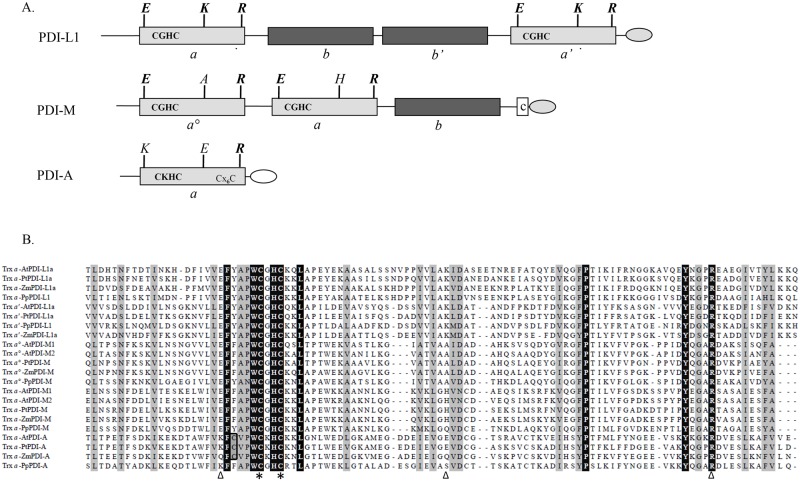
Sequence characteristics and domain organisation of characterized poplar PDI isoforms and their plant orthologs. A. Modular organisation of poplar PDI-L1a, PDI-M and PDI-A. Boxes in light grey and dark grey indicate redox active (*a°*, *a’* or *a*) and inactive (*b* or *b’*) Trx modules, respectively. Additional letters indicate the amino acids important for the redox properties. The presence of classical (KDEL) ER retention signals are represented in a light grey egg-shaped form. The unusual DK[D/E]L C-terminal sequence found in PDI-A is represented in white. B. Amino acid sequence alignment of the catalytic *a* modules from PDI-L1a, PDI-M and PDI-A isoforms belonging to At, *Arabidopsis thaliana* (AtPDI-A, At1g07960; AtPDI-L1a, At1g21750; AtPDI-M1, At1g04980; AtPDI-M2, At2g32920); Pp, *Physcomitrella patens* (PpPDI-A, Phpat.006G010400; PpPDI-L1, Phpat.015G023600; PpPDI-M, Phpat.004G043700); Pt, *Populus trichocarpa* (PtPDI-A, Potri.009G004500; PtPDI-L1a, Potri.002G082100; PtPDI-M, Potri.014G160000) and Zm, *Zea mays* (ZmPDI-A, GRMZM2G073628; ZmPDI-L1a, GRMZM2G091481; ZmPDI-M, GRMZM2G389173). The Trx modules were delimited according to Pfam only database (http://pfam.sanger.ac.uk/) and the alignment was built using ClustalW algorithm at the NPSA web portal (http://npsa-pbil.ibcp.fr). Output of this alignment was made with the ESPript web portal (http://espript.ibcp.fr/ESPript/cgi-bin/ESPript.cgi). Amino acids strictly conserved appear in black whereas partially conserved amino acids are indicated in light grey. Cysteines of the active site signature are indicated with stars, The E, K, R residues also represented in the panel A and possibly involved in the modulation of the cysteine pKa are indicated by triangles.

In order to analyze the biochemical properties of PDI with different domain organisations, three poplar isoforms referred to as PDI-L1a, PDI-M and PDI-A have been selected for an in-depth analysis [5]. PDI-L1a and PDI-M are multi-modular proteins composed of 4 and 3 domains respectively but both proteins possess two *a* domains containing the conventional WCGHC active site signature (Fig 1A and 1B). On the contrary, PDI-A exhibits a single *a* domain with a modified WCKHC signature, which is also found in land plant orthologs. It is interesting to note that ancestral PDI-A relatives found in *Selaginella moellendorffii* and *Physcomitrella patens* have the regular WCGHC signature.

Besides active site residues, there are 5 additional strictly conserved residues, a Phe and a Leu at positions -5 and +6 compared to the catalytic Cys, the *cis*-Pro conserved in all oxidoreductases having a Trx fold, and a Tyr and an Arg in the C-terminal part. This Arg was proposed to modulate the pKa of the C-terminal active site Cys, necessary for the PDI reoxidation step, by moving into and out of the active site, depending on the catalytic step [32]. Two other residues, a Glu and a Lys, highlighted in Fig 1A and 1B, are involved in the modulation of the N-terminal active site cysteine pKa at least in *E*. *coli* Trx1 [33]. Whereas they are strictly conserved in poplar PDI-L1a (E_52_/K_86_ and E_397_/K_431_), only the Glu is conserved in the two *a* domains of PDI-M, the Lys being replaced by an Ala (A_85_) or a His (H_213_) in the *a°* and *a* modules respectively. None of them is conserved in PDI-A. All these observations raise the question of whether the redox properties of the atypical PDI-A and PDI-M are influenced by these variations in sequence and domain organisation.

### Among atypical PDI, PDI-A is inactive and PDI-M is less efficient than the regular PDI-L1a

**Fig 2:**
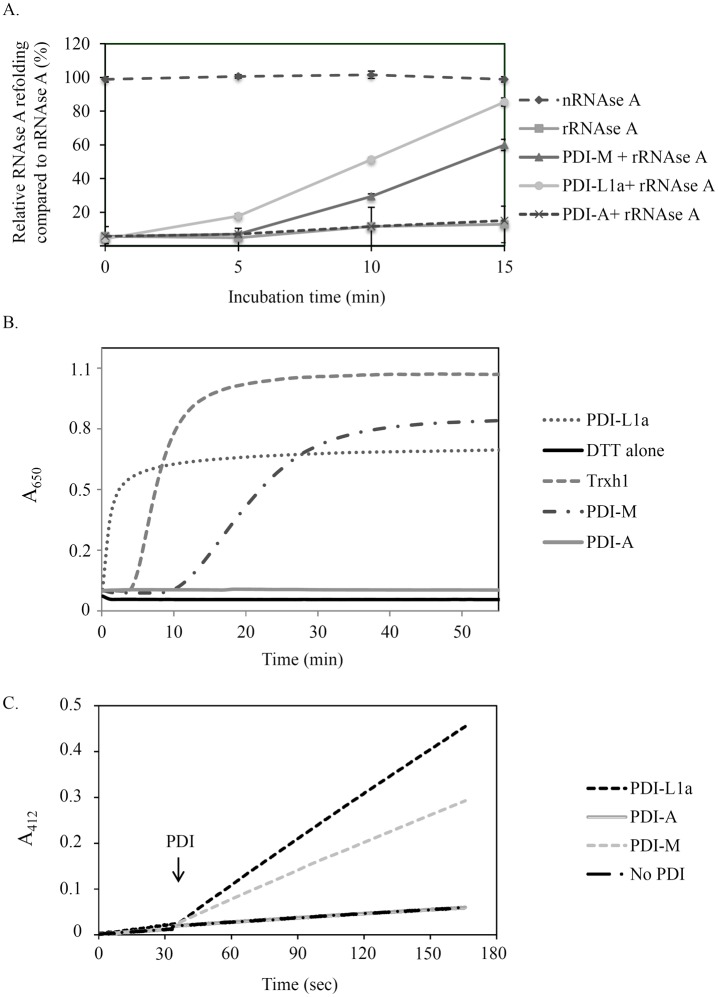
Comparative analysis of the oxidoreductase activities of poplar PDI isoforms. A. Oxidative refolding of RNase A. Results are represented as percentages of activity relative to the activity of native RNase A (nRNase A). The reduced RNase A (rRNase A) was tested alone or upon incubation with PDI-L1a, PDI-M or PDI-A for the indicated times. Measurements were made in triplicate and error bars indicate standard deviation. B. Insulin reduction. It was assessed by measuring the turbidity at 650 nm caused by the precipitation of insulin upon reduction with DTT alone or supplemented with 5 μM PDI-L1a, PDI-M, PDI-A or Trx h1. The reaction curve shown is representative of at least three independent repetitions. C. PDI reduction by AtNTRB. DTNB reduction was followed at 412 nm in the presence of NADPH, AtNTRB alone or with PDI-L1a, PDI-M or PDI-A. The PDI have been added after 30 seconds. The reaction curve shown is representative of at least three independent repetitions.

After producing in E. *coli* all three proteins devoid of their putative N-terminal ER targeting sequences and purifying them to homogeneity, the classical test used for measuring PDI activity *i*.*e*., the oxidative refolding of a reduced and denatured RNase A, has been used for an initial comparison of their respective activity. PDI-L1a was the most efficient protein, as, after an incubation time of 15 min, it allowed the recovery of *ca* 90% of the activity relatively to the one observed with native RNase A (Fig 2A). At the same time, RNase A activity recovery was only 60% in the presence of PDI-M whereas the results obtained with PDI-A were similar to the reaction achieved by omitting PDI, indicating that the latter did not promote oxidation reactions, at least with RNase A.

Then, the capacity of all these PDI to catalyze the reduction of disulfide bridges instead of promoting their formation was analyzed by measuring insulin reduction, which is accompanied by its precipitation as measured by recording absorbance at 650 nm. In this test, PDI-L1a was the most efficient protein, even more than poplar Trx h1, with the reduction starting after 0.5 and 4 min respectively at concentrations of 5 μM (Fig 2B). PDI-M can also perform insulin reduction but, as in the previous assay, it was less efficient than PDI-L1a or Trx h1. PDI-A was not able to reduce insulin at all. Considering the presence of two catalytic domains in PDI-L1a and PDI-M, the better reducing activity of PDI-L1a compared to PDI-M could suggest that both catalytic domains of PDI-L1a possess reductase activity whereas only one PDI-M domain may be efficient. Moreover, independently of the rate of the reaction, the fact that the turbidity was lower is the case of both PDI compared to Trx h1 might indicate that not all insulin disulfide bonds are reduced by PDI.

Finally, since a variety of PDI can be efficiently reduced by a NADPH/NADPH thioredoxin reductase (NTR) system, we examined whether PDI can be reduced by the *A*. *thaliana* NTRB isoform using DTNB as an electron acceptor. Although this is presumably not the physiological reductant of ER-targeted PDI, PDI-A does not have the typical ER-retention signal contrary to PDI-L1a and PDI-M and such a system would be extremely convenient afterwards for measuring the reducing capacity of PDI by using usual Trx targets. As in the other assays, PDI-L1a was more efficient than PDI-M and PDI-A was inactive (Fig 2C).

### Atypical PDI have redox midpoint potentials adequate for an oxidoreductase activity

**Fig 3:**
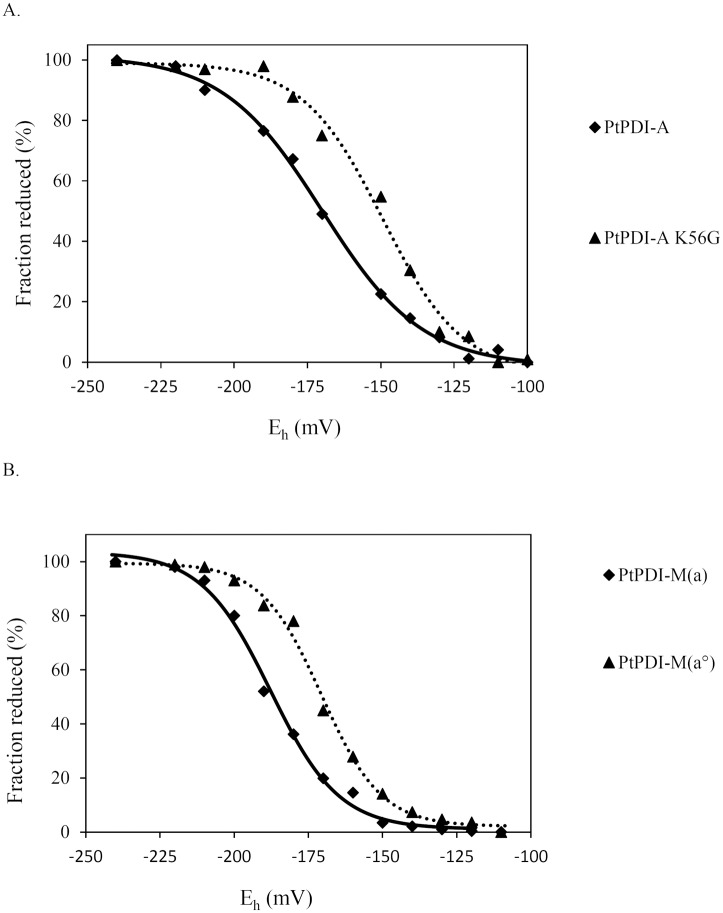
Redox midpoint potential of the catalytic domains of poplar PDI-A and PDI-M. A. Redox midpoint potential of PDI-A (solid line) and PDI-A K56G (dotted line). B. Redox midpoint potential of the *a* (solid line) and *a°* (dotted line) modules of PDI-M measured using the PDI-M C36/39S and PDI-M C165/168S variants respectively. The titrations were carried out using a total glutathione concentration of 2 mM in the redox buffer and with a redox equilibration time of 2 h, before labelling free protein thiols by mBBr.

Besides structural considerations, the redox properties of oxidoreductases are also governed by the pKa of the catalytic cysteines and the redox potential of the catalytic disulfides. Since PDI-A was inactive in all assays and possessed a particular active site signature and differences are observed among PDI, we sought to evaluate the redox potentials of both atypical PDI, PDI-A and PDI-M. It is well documented that the nature of the spacing residues between the two cysteine residues influences the redox potential values of proteins of the Trx superfamily [34, 35]. Hence, for PDI-A, a K56G variant, the active site sequence of which was changed from WCKHC to the regular WCGHC signature, has been produced and characterized. The E_m_ value of PDI-A was determined to be -170 mV ± 5 mV whereas the one measured for PDI-A K56G was -150 mV ± 5 mV (Fig 3A). However, the observed 20 mV increase for the PDI-A K56G variant did not allow acquiring oxidoreductase activity since it was also inactive in all assays used previously (RNase A refolding, insulin and DTNB reduction) (data not shown).

For PDI-M, the redox potentials of the disulfide formed by the cysteine residues found in both *a* domains was measured after producing two variants corresponding to the full-length protein but where the dicysteinic WCGHC motif of each *a* domain was substituted by a WSGHS motif. Using PDI-M C165/168S and PDI-M C36/39S variants, E_m_ values of -170 mV ± 5 mV and of -190 mV ± 5 mV have been determined for the catalytic disulfides found in the *a°* and *a* domains respectively (Fig 3B). The PDI-M C165/168S variant will be thereafter referred to as PDI-M(*a°*) since only the *a°* domain possesses cysteines susceptible to provide the oxidoreductase activity and the PDI-M C36/39S variant as PDI-M(*a*) for the same reasons.

### PDI-A incorporates a [Fe_2_S_2_] cluster upon expression in *E*. *coli*

**Fig 4:**
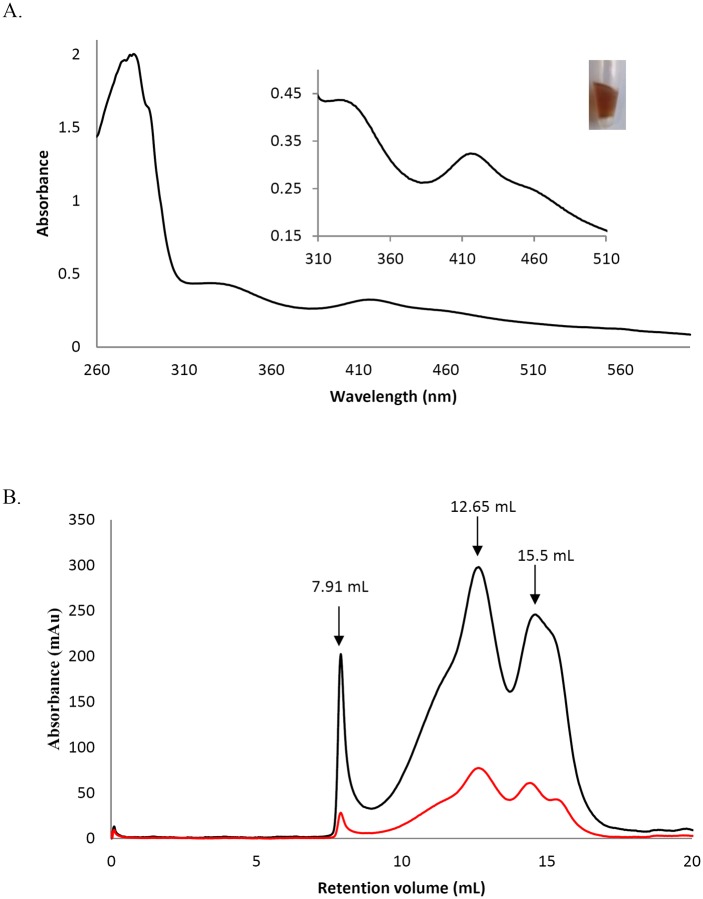
The recombinant PDI-A binds an Fe-S cluster. A. UV-visible absorption spectrum between 260 and 600 nm of anaerobically purified PDI-A. The blow-up shows absorbance bands between 310 and 510 nm characteristics of a [Fe_2_-S_2_] cluster and a picture of a tube containing the protein in its holoform. B. Analytical size-exclusion chromatography performed on a Sephadex S75 10–300 column using 100 μg and a 30mM Tris-HCl pH 8.0, 200 mM NaCl buffer. The black curve corresponds to the absorbance at 280 nm and the red curve at 420 nm.

The absence of activity for PDI-A was intriguing. By comparing *E*. *coli* cell pellets after centrifugation, we observed a darker colour for the one expressing PDI-A. For this reason, the protein was purified under anaerobic conditions. The UV-visible absorption spectrum of the purified brownish protein exhibited two peaks around 320 and 420 nm and a shoulder at 460 nm which are likely characteristic of the presence of an iron-sulfur (Fe-S) cluster of the [Fe_2_S_2_] type (Fig 4A). The analytical measurement of iron and sulfide contents revealed 1.28 ± 0.13 Fe atoms and 1.10 ± 0.05 S atoms per monomer. Analytical gel filtration experiments showed a small part of aggregated protein and two major, not well separated, peaks with elution volumes at 7.91, 12.65 and 15.5 ml respectively (Fig 4B). Considering the theoretical molecular mass of PDI-A of *ca* 15 kDa, the peak at 12.65 ml corresponding to an apparent volume of *ca* 115 kDa may be interpreted as an octameric form and the one at 15.5 ml corresponding to an apparent volume of *ca* 27 kDa may be interpreted as a dimeric form. Both peaks contained PDI-A with Fe-S cluster as attested by the presence of the absorbance at 420 nm. Altogether, although, PDI-A seems to exist under various oligomeric forms, these results could be consistent with PDI-A binding a [Fe_2_S_2_] cluster into a dimer. The association of dimers may lead to this higher oligomeric form. Further spectroscopic analyses are however needed for a definitive assessment of the type(s) of cluster that PDI-A can incorporate.

### The two catalytic domains of PDI-M do not possess equivalent oxidoreductase properties

In subsequent experiments, we focused our effort on the atypical PDI-M. Using PDI-M(*a°*) and PDI-M(*a*) variants, we have investigated the contribution of each catalytic domain to the PDI-M redox properties using the same assays *i*.*e*., RNase A oxidative refolding, DTNB and insulin reduction, but also by testing activities typical of Trx *i*.*e*., the capacity to activate the chloroplastic NADP-MDH, a well-characterized redox-regulated chloroplastic protein catalyzing the conversion of oxaloacetate to malate, and to sustain the peroxidase activity of a chloroplastic Prx belonging to the 2-Cys Prx subgroup by regenerating the reduced active form of the protein.

**Fig 5:**
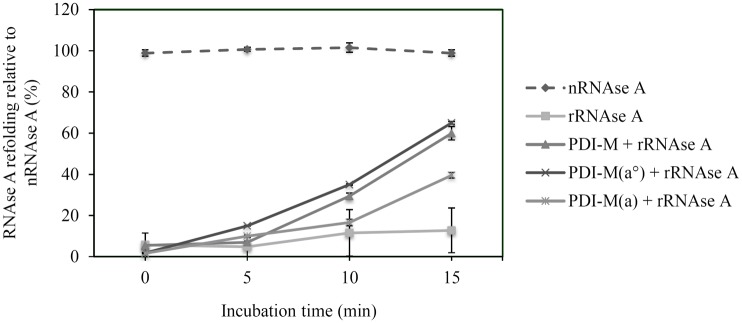
Oxidative refolding of reduced RNase A by the PDI-M catalytic domains. Results are represented as percentages relative to native RNase A (nRNase A) for the indicated time. The activity of the reduced and denatured RNase A (rRNase A) was tested alone or after incubation with PDI-M, PDI-M(*a°*) or PDI-M(*a*). Measurements were made in triplicate and error bars indicate standard deviation.

In the RNase A refolding assay, PDI-M(*a°*) presented an efficiency comparable to the one of PDI-M whereas PDI-M(*a*) exhibited only 50 to 60% of this activity after 15 minutes (Fig 5). This suggested that the *a°* module is likely the major contributor for the oxidase activity measured with the intact protein and that there may be a partial compensation by the second domain in the absence of the catalytic cysteines of the *a°* module.

**Fig 6:**
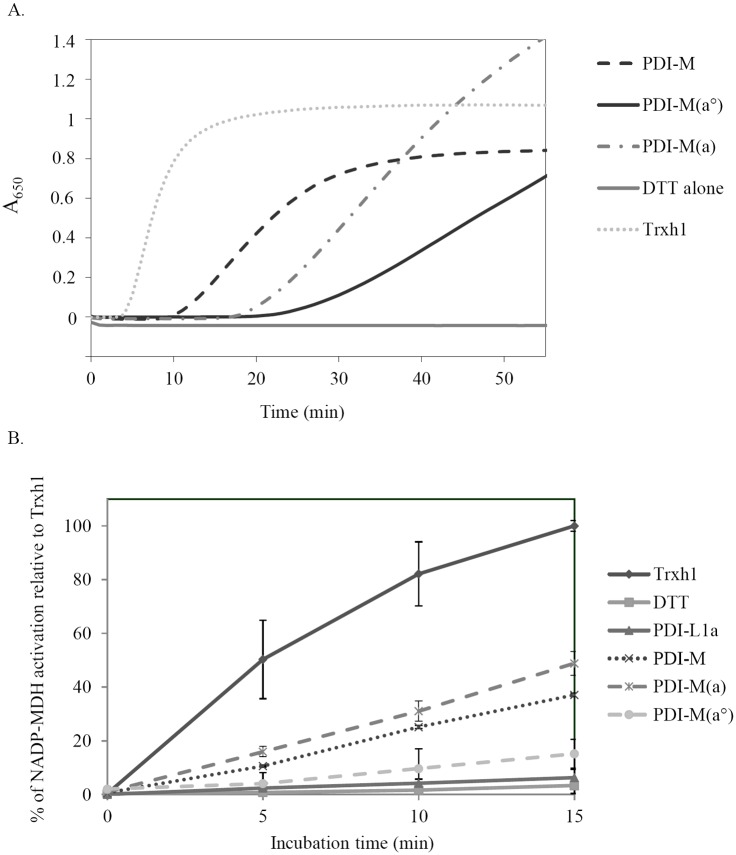
Reductase activity of the PDI-M catalytic domains. A. Insulin reduction was assayed by measuring the turbidity at 650 nm caused by its precipitation upon reduction using DTT alone or in the presence of 5 μM Trx h1, PDI-M, PDI-M(*a°*), PDI-M(*a*). As in Figs 3 and 5, PDI-M(*a°*) and PDI-M(*a*) refers to the PDI-M C165/168S and PDI-M C36/39S variants respectively. B. Activation of NADP-malate dehydrogenase. The NADP-MDH activity was followed by measuring the NADPH-coupled oxaloacetate conversion at 340 nm after a pre-incubation of the purified recombinant protein with DTT alone (solid line, squares) or in the presence of Trx h1 (solid line, diamonds), PDI-L1a (solid line, triangles) PDI-M (dotted line, crosses), PDI-M(*a°*) (dashed line, circles) and PDI-M(*a*) (dashed line, crosses) for the indicated time. The results have been represented as percentages relative to the maximal reactivation obtained with Trxh1 after 15 min. Measurements were made in triplicate and error bars indicate standard deviation.

Then, the reductase activity of the respective PDI-M domains was tested by assessing their capacity to reduce insulin and to activate NADP-MDH in the presence of DTT. In the insulin reduction assay, PDI-M(*a*) was more efficient than PDI-M(*a°*) (Fig 6A). In this case, the fact that the activity of both domains was decreased compared to the activity of PDI-M suggests that both domains contribute to the reductase activity of PDI-M although with different efficiencies. In the NADP-MDH activation assay, we observed that PDI-M was the only PDI representative able to activate the enzyme since PDI-A (data not shown) and PDI-L1a were unable to do that (Fig 6B). This was quite surprising taking into account the unfavourable thermodynamic barrier, NADP-MDH possesses disulfides with very negative redox potential, around -300 mV at pH 7.0 [36]. However, the observed activation was less efficient compared to the one observed at identical concentrations with Trx h1 used as a reference. The two PDI-M domains presented different reactivities toward oxidized NADP-MDH, PDI-M(*a*) was even slightly more active than PDI-M whereas PDI-M(*a°*) was less active (Fig 6B). Altogether, these data indicated that the *a* module is a more efficient reductant than the *a°* module.

**Fig 7:**
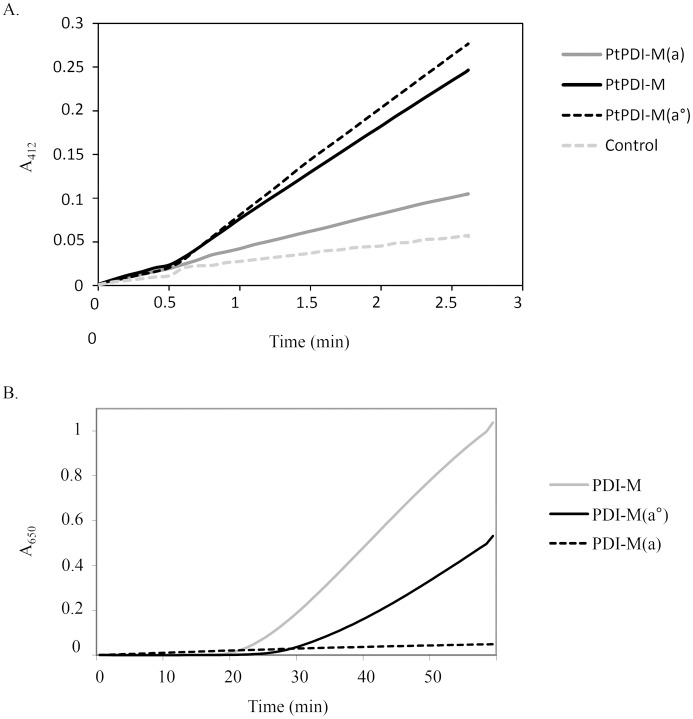
Reduction of the PDI-M catalytic domains by AtNTRB as assayed using DTNB and insulin. A. DTNB reduction assay. Representative kinetic curves obtained in the presence of NADPH and AtNTRB alone or with 0.2 μM PDI-M, PDI-M(*a°*) or PDI-M(*a*). B. Insulin reduction assessed using NADPH/AtNTRB as an electron donor system and 5 μM of PDI-M, PDI-M(*a°*) or PDI-M(*a*).

The properties of PDI-M domains have been further examined using the DTNB assay and AtNTRB as an electron donor. The PDI-M(*a°*) domain was as efficient as PDI-M whereas PDI-M(*a*) was only very poorly able to promote DTNB reduction (Fig 7A). To check whether this was due to the inability to be reduced by NTR or to reduce DTNB, insulin reduction assays have been performed with the NADPH/NTR system as the electron supplier instead of DTT (Fig 7B). The absence of activity for PDI-M(*a*), which was in fact the most efficient for insulin reduction when DTT was used as a reductant, obviously indicates that the *a* domain is not or only poorly reduced by NTR and that the catalytic disulfides of PDI-M are asymmetrically targeted by NTR. On the other hand, insulin reduction by PDI-M(*a°*) was less efficient than by PDI-M (Fig 7B). Since PDI-M(*a°*) and PDI-M had comparable efficiency for DTNB reduction, these results indicate that mutating the active site cysteines of the *a* domain may somehow affect the redox properties of the *a°* module (possibly at the level of the 3D structure or of substrate recognition (here insulin)) which incidentally suggests that these two adjacent domains are connected.

**Fig 8:**
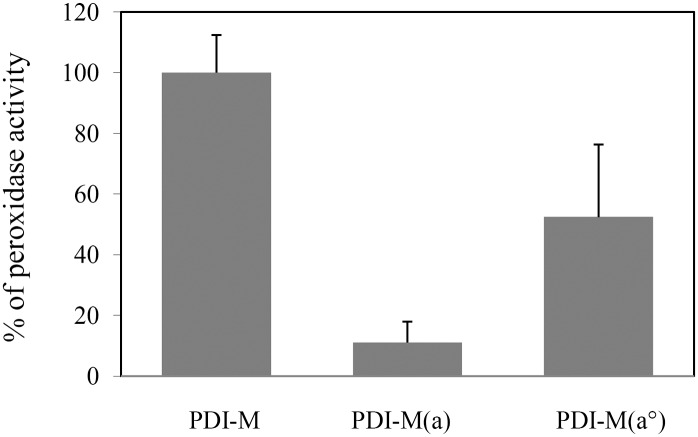
*In vitro* recycling of 2-Cys Prx by PDI-M. The peroxidase activity of *A*. *thaliana* 2-Cys Prx was assessed using a coupled assay measuring NADPH oxidation. Results obtained for PDI-M(*a°*) or PDI-M(*a*) are represented as percentages relative to the activity obtained with PDI-M fixed at 100%. Measurements were made in triplicate and error bars indicate standard deviation.

Finally, we have taken advantage of the NADPH-coupled NTR reduction system to examine the reduction of Prx by PDI-M. Indeed, it was shown recently that an ER-targeted human 2-Cys Prx (PrxIV) can accept electrons from two PDI isoforms, P5 and ERp46 [37, 38]. Although there is no evidence yet that a 2-Cys Prx is targeted to the ER in plants, we tested the biochemical capability of PDI-M to reduce the chloroplastic *Arabidopsis* 2-Cys Prx (At2-Cys Prx). As shown in Fig 8, PDI-M promoted the regeneration of At2-Cys Prx in the presence of the NADPH-NTR system as an electron donor. As expected from its poor reduction by AtNTRB, the PDI-M(*a*) was almost inefficient, only *ca* 10% activity was retained compared to PDI-M. The PDI-M(*a°*) retained about 50% activity. Such a decrease may again indicate that having mutated the active site cysteines of the *a* module impacted the *a°* module reactivity.

## Discussion

Protein disulfide isomerases are versatile proteins catalyzing the reduction, formation and/or isomerisation of disulfide bonds *in vitro* and also possibly *in vivo* depending on the substrates, on their subcellular localizations and/or on the cellular and subcellular redox potentials. In connection with these elements and with the variety of substrates, there are multiple PDI with variable module arrangements which provide the necessary flexibility in the PDI activity pattern [39–41]. For multi-modular proteins, each domain could ensure a “specific” function. As an example, the *b’* module of mammalian ERp57 or PDI proteins, which is redox inactive, is essential for substrate or chaperone partner recruitment [13, 15, 42, 43]. By comparing the biochemical properties of plant PDI belonging to three different classes and with different modular organisation, we also brought elements indicating that the catalytic domains within a given PDI or between PDI do not have the same redox properties.

### What is the biochemical function of the single module PDI-A?

Using the battery of substrates tested, we did not detect any oxidase or reductase activity for the atypical mono-module PDI-A, which raises the question of its physiological role and partners. First, considering the DKDL C-terminal sequence, divergent from classical ER retention signals (very often KDEL or KEEL), the question of its subcellular localization is still open. It is worth mentioning that human ERp18, a small mono-domain PDI was shown to exhibit an oxidase activity on synthetic peptides [44]. However, despite their comparable modular organisation, PDI-A and ERp18 might not be true orthologs, exhibiting only *ca* 15% sequence identity. Proteins involved in oxidation reactions usually exhibit redox potentials ranging from less than -100 mV for some prokaryotic DsbA proteins to -180 mV for the *a* modules of classical PDI [41, 45, 46]. Hence, the E_m_ value of -170 mV determined for PDI-A is clearly compatible with oxidation reactions and should not be the reason for the absence of activity. So, why is PDI-A inactive? A first possible explanation could be that the transit peptide has been cleaved at an unfavourable position, eliminating some N-terminal residues essential for activity. However, this seems unlikely since we have cleaved only 26 amino acids in a region which is not conserved and without predicted secondary structure and the protein is fully soluble. Hence, the absence of reactivity is more likely linked to specific differences in the sequence of PDI-A compared to other PDI.

First, the CVPWCKHC active site signature which is conserved in PDI-A orthologs replaces the usual [Y/F]APWCGHC (Fig 1). We initially thought that the presence of a charged residue between the two cysteines might be strongly unfavourable. However, we have observed that the atypical WCKHC, although influencing the redox midpoint potential, was not responsible for the absence of activity. In fact, there are many other sequence variations that would explain the lack of oxidoreductase activity. First, the FA or YA pair, found in the above-mentioned signature and usually quite conserved in PDI, is replaced by a CV pair. Then, among the three residues (Glu, Lys, Arg, highlighted in Fig 1) susceptible of contributing to the lowering the pKa of the catalytic cysteines in some Trxs, only the Arg is present in PDI-A [32, 33, 47]. The two other residues (Glu, Lys) are replaced by residues with an opposite charge (Lys, Glu) in PDI-A (Fig 1A). From its absence in PDI-M and the work performed on *E*. *coli* Trx1, the Lys does not seem to be as important as the Glu and might thus not be absolutely required [33]. Besides these sequence variations, it is still possible that we have not used proper substrates yet in our *in vitro* assays and that this protein possesses highly specific physiological partners. Alternatively, it may have evolved to play a completely different function, unrelated to dithiol-disulfide exchanges. The observation that PDI-A can incorporate a [Fe_2_S_2_] cluster was totally unexpected because this has to our knowledge never been observed for a PDI member. It appears a bit less surprising when taking into account that proteins sharing the same thioredoxin fold, *i*.*e*. some thioredoxins or glutaredoxins, including the ER-targeted GRX6 isoform of *S*. *cerevisiae*, are able to do this [48–51]. However, the physiological relevance of this observation is unclear and it remains to be investigated whether such cluster is not an artefact linked to *E*. *coli* expression and can indeed be formed in plant cells. It would also be important to determine the subcellular localization of the protein, because Fe-S cluster maturation systems are only found in organelles and in the cytosol, not in the ER [52].

### Does the domain organisation of PDI confer them different biochemical properties?

The second aim of the study was the biochemical characterization of a poplar PDI-M isoform possessing an *a°-a-b* domain organisation quite different from the *a-b-b’-a’* of the classical and well-characterized PDI-L. In particular, the organisation of the catalytic domains in tandem raises the question of their respective contribution to protein reactivity and of their possible connectivity. The latter point is in fact one of the unsolved questions for most PDI, although such cooperativity has been proposed based on the U shape arrangement of the four Trx domains of the yeast PDI1p, very similar to DsbC or DsbG [41]. We have compared the PDI-L1a and PDI-M capacity to reduce insulin and NADP-MDH or to oxidize NTR and RNase-A. It was previously reported that some PDI can act as oxidants of NTR or in other words that NTR can reduce oxidized PDI [53].

Except for the NADP-MDH activation assay, where it was not active at all, PDI-L1a was always more efficient than PDI-M in all other assays measuring reductase or oxidase activities. This difference of activity observed with poplar proteins is similar to what was recently observed with rice orthologs, indicating that this may be a general feature of these two classes [40]. The better efficiency of PDI-L1a, in particular in reduction assays, may be explained by the strict conservation of the charged amino acids required for decreasing the pKa of the N-terminal catalytic cysteine. Indeed, both *a* modules of PDI-L1a possess the usual Glu and Lys residues, whereas both domains of PDI-M contain the Glu but an Ala and a His in the *a°* and *a* modules respectively (Fig 1). That PDI-L1a was inactive towards NADP-MDH whereas PDI-M exhibited noticeable activities could possibly derive from a natural tendency to interact with non-folded or folded proteins respectively. Indeed, it was shown for mammalian enzymes that PDI-L interact directly with misfolded or unfolded proteins via the mediation of a hydrophobic patch in the *b’* module, whereas P5 proteins, the mammalian PDI-M orthologs, are partners of the BiP chaperone, a folded protein, which is thought to bring mis-oxidized/misfolded proteins to P5 for their correct oxidative folding [54]. Hence, by analogy and despite a lower “intrinsic” reductase activity compared to PDI-L1a, PDI-M could interact with and promote the activation of native proteins such as NADP-MDH. In agreement with the difference observed in their *in vitro* biochemical properties, it was shown by genetic studies that a rice PDI-M ortholog, OsPDI2.3, and a rice PDI-L ortholog, PDIL1.1 do not have redundant functions in the ER, having for instance different storage proteins as substrates in the endosperm [21].

These dual and opposite oxidase/reductase activities observed *in vitro* raise the question of their physiological relevance and occurrence. The *in vivo* measurement of the redox state of PDI in HEK-293 cells showed for instance a mixture of fully reduced (50%), fully oxidized (16%) and partially oxidized forms of the *a* or *a’* modules (18/15%) [55]. These results indicate first that neither of the two domains of this human PDI might exclusively catalyze substrate oxidation or reduction, but also that the protein redox state may be adequate for both reactions. This potential dual function has been nicely illustrated recently by demonstrating that several PDI can reduce ER-resident proteins as 2-Cys Prx (PrxIV) or vitamin K epoxide reductase [37, 56–58]. This alternative route of PDI oxidation which would occur independently of ERO1 protein *i*.*e*., without producing H_2_O_2_, should clearly decrease reactive oxygen species formation in the ER [59].

### Are there specific contributions and/or connections between the catalytic domains in multi-domain PDI?

It is documented that the *a’* module (the C-terminal one) of a human regular PDI-L, which presents the highest redox potential (*ca* -150 mV), possesses a preponderant oxidase activity toward reduced and scrambled RNase A [41]. Also, it was shown that only one of the catalytic disulfides of this PDI is directly oxidized by Ero1α and with a slow turnover rate [39]. With the above-mentioned evidence showing the complexity of assessing and interpreting the *in vivo* redox state [55], these elements suggest that the presence of two catalytic *a* domains might be important for the formation of native disulfides in proteins entering the secretory pathway by ensuring either oxidase or isomerase reactions.

Although experiments have not been achieved with poplar PDI-L1a, we have observed such a duality among the *a* domains in PDI-M. The *a* module, having the cysteine pairs with the lowest redox potential (-170 mV) is the most efficient for reduction reactions (insulin, NADP-MDH) (Figs 6 and 7). On the contrary, the *a°* module, having the cysteine pairs with the highest redox potential (-150 mV), is more efficient for oxidation reaction than the *a* module, promoting the recovery of RNase A activity similar to PDI-M levels. Accordingly, the *a°* module of orthologs (rice PDI2,3 and mammalian P5) is also the major contributor in the oxidative refolding of reduced or scrambled RNase A [40, 60]. In line with this observation, the *a°* module can efficiently oxidize NTR contrary to the *a* module. The incapacity of the *a* module to oxidize NTR or RNase A may relate to steric hindrance or unfavourable protein/protein interactions rather than to thermodynamic factors, the redox potentials of both domains being still rather close. The structure of the *E*. *coli* NTR-Trx complex indicated that Trx interacts with NTR mostly via hydrophobic residues (W33, I60, G74 and I75) and via R73 [61]. By comparing all *a* domains of PDI-L1a and PDI-M, it is interesting to note that two of these five residues differ in the *a* domain of PDI-M compared to the three other *a* domains analyzed where they are all strictly similar (not shown). Hence, it is tempting to attribute the inability of the *a* domain of PDI-M to interact with NTR to these changes. Another possible explanation could rely on steric factors. In PDI-L1a, if it adopts a regular structure, the two *a* domains should be found at the extremity of the U-shape structure and thus be very likely accessible to NTR. On the contrary, the fact that the two *a* domains in PDI-M are adjacent and that the *a* domain is sandwiched between the *a°* and *b* domains may be problematic for the accessibility of NTR which is an homodimer of two identical 35 kDa subunits.

In the course of these experiments and taking advantage of the fact that the *a°* module of PDI-M is reduced by AtNTRB whereas the *a* module is not or very poorly, we have observed some sort of cross-talk or interference between both domains. Indeed, PDI-M(*a°*) is as efficient as PDI-M in the assay with the small DTNB molecule (Fig 7A) whereas it is much less efficient with insulin and 2-Cys Prx, retaining about 50% activity (Fig 7B and 7C). Thus, it may be that the presence of a redox active *a* module is needed for a maximal efficiency of the *a°* module with some substrates. Alternatively, introducing cysteine substitutions in the *a* module may have generated subtle conformational changes which hamper recognition of large substrates by the *a°* module. These substitutions may have more impact in PDI-M because the two catalytic domains are adjacent.

## References

[1] Grimshaw JP, Stirnimann CU, Brozzo MS, Malojcic G, Grutter MG, Capitani G, et al DsbL and DsbI form a specific dithiol oxidase system for periplasmic arylsulfate sulfotransferase in uropathogenic *Escherichia coli*. J Mol Biol. 2008;380(4):667–80. 10.1016/j.jmb.2008.05.031 18565543

[2] Inaba K. Disulfide bond formation system in *Escherichia coli*. J Biochem. 2009;146(5):591–7. 10.1093/jb/mvp102 19567379

[3] Arts IS, Gennaris A, Collet JF. Reducing systems protecting the bacterial cell envelope from oxidative damage. FEBS Lett. 2015;589(14):1559–68. 10.1016/j.febslet.2015.04.057 25957772

[4] Depuydt M, Leonard SE, Vertommen D, Denoncin K, Morsomme P, Wahni K, et al A periplasmic reducing system protects single cysteine residues from oxidation. Science. 2009;326(5956):1109–11. 10.1126/science.1179557 19965429

[5] Selles B, Jacquot JP, Rouhier N. Comparative genomic study of protein disulfide isomerases from photosynthetic organisms. Genomics. 2011;97(1):37–50. 10.1016/j.ygeno.2010.10.001 20951197

[6] Alon A, Grossman I, Gat Y, Kodali VK, DiMaio F, Mehlman T, et al The dynamic disulphide relay of quiescin sulphydryl oxidase. Nature. 2012;488(7411):414–8. 10.1038/nature11267 22801504 PMC3521037

[7] Kodali VK, Thorpe C. Oxidative protein folding and the Quiescin-sulfhydryl oxidase family of flavoproteins. Antioxid Redox Signal. 2010;13(8):1217–30. 10.1089/ars.2010.3098 20136510 PMC2959182

[8] Okuda A, Matsusaki M, Higashino Y, Masuda T, Urade R. Disulfide bond formation activity of soybean quiescin sulfhydryl oxidase. Febs J. 2014;281(23):5341–55. 10.1111/febs.13079 25265152

[9] Riemer J, Bulleid N, Herrmann JM. Disulfide formation in the ER and mitochondria: two solutions to a common process. Science. 2009;324(5932):1284–7. 10.1126/science.1170653 19498160

[10] Collet JF, Messens J. Structure, function, and mechanism of thioredoxin proteins. Antioxid Redox Signal. 2010;13(8):1205–16. 10.1089/ars.2010.3114 20136512

[11] Pedone E, Limauro D, D'Ambrosio K, De Simone G, Bartolucci S. Multiple catalytically active thioredoxin folds: a winning strategy for many functions. Cell Mol Life Sci. 2010;67(22):3797–814. 10.1007/s00018-010-0449-9 20625793 PMC11115506

[12] Wang C, Yu J, Huo L, Wang L, Feng W, Wang CC. Human protein-disulfide isomerase is a redox-regulated chaperone activated by oxidation of domain a'. J Biol Chem. 2012;287(2):1139–49. 10.1074/jbc.M111.303149 22090031 PMC3256865

[13] Yagi-Utsumi M, Satoh T, Kato K. Structural basis of redox-dependent substrate binding of protein disulfide isomerase. Sci Rep. 2015;5:13909 10.1038/srep13909 26350503 PMC4563560

[14] Appenzeller-Herzog C, Ellgaard L. The human PDI family: versatility packed into a single fold. Biochim Biophys Acta. 2008;1783(4):535–48. 10.1016/j.bbamcr.2007.11.010 18093543

[15] Kozlov G, Maattanen P, Thomas DY, Gehring K. A structural overview of the PDI family of proteins. Febs J. 2010;277(19):3924–36. 10.1111/j.1742-4658.2010.07793.x 20796029

[16] Houston NL, Fan C, Xiang JQ, Schulze JM, Jung R, Boston RS. Phylogenetic analyses identify 10 classes of the protein disulfide isomerase family in plants, including single-domain protein disulfide isomerase-related proteins. Plant Physiol. 2005;137(2):762–78. 10.1104/pp.104.056507 15684019 PMC1065376

[17] Iwasaki K, Kamauchi S, Wadahama H, Ishimoto M, Kawada T, Urade R. Molecular cloning and characterization of soybean protein disulfide isomerase family proteins with nonclassic active center motifs. Febs J. 2009;276(15):4130–41. 10.1111/j.1742-4658.2009.07123.x 19583593

[18] Kamauchi S, Wadahama H, Iwasaki K, Nakamoto Y, Nishizawa K, Ishimoto M, et al Molecular cloning and characterization of two soybean protein disulfide isomerases as molecular chaperones for seed storage proteins. Febs J. 2008;275(10):2644–58. 10.1111/j.1742-4658.2008.06412.x 18422652

[19] Wadahama H, Kamauchi S, Ishimoto M, Kawada T, Urade R. Protein disulfide isomerase family proteins involved in soybean protein biogenesis. Febs J. 2007;274(3):687–703. 10.1111/j.1742-4658.2006.05613.x 17181539

[20] Onda Y, Kumamaru T, Kawagoe Y. ER membrane-localized oxidoreductase Ero1 is required for disulfide bond formation in the rice endosperm. Proc Natl Acad Sci U S A. 2009;106(33):14156–61. 10.1073/pnas.0904429106 19666483 PMC2729036

[21] Onda Y, Nagamine A, Sakurai M, Kumamaru T, Ogawa M, Kawagoe Y. Distinct roles of protein disulfide isomerase and P5 sulfhydryl oxidoreductases in multiple pathways for oxidation of structurally diverse storage proteins in rice. Plant Cell. 2011;23(1):210–23. 10.1105/tpc.110.079509 21278127 PMC3051231

[22] Wadahama H, Kamauchi S, Nakamoto Y, Nishizawa K, Ishimoto M, Kawada T, et al A novel plant protein disulfide isomerase family homologous to animal P5—molecular cloning and characterization as a functional protein for folding of soybean seed-storage proteins. Febs J. 2008;275(3):399–410. 10.1111/j.1742-4658.2007.06199.x 18167147

[23] Kim J, Mayfield SP. The active site of the thioredoxin-like domain of chloroplast protein disulfide isomerase, RB60, catalyzes the redox-regulated binding of chloroplast poly(A)-binding protein, RB47, to the 5' untranslated region of psbA mRNA. Plant Cell Physiol. 2002;43(10):1238–43. 12407204 10.1093/pcp/pcf129

[24] Yang P, Lupken T, Habekuss A, Hensel G, Steuernagel B, Kilian B, et al PROTEIN DISULFIDE ISOMERASE LIKE 5–1 is a susceptibility factor to plant viruses. Proc Natl Acad Sci U S A. 2014;111(6):2104–9. 10.1073/pnas.1320362111 24481254 PMC3926060

[25] Couturier J, Didierjean C, Jacquot JP, Rouhier N. Engineered mutated glutaredoxins mimicking peculiar plant class III glutaredoxins bind iron-sulfur centers and possess reductase activity. Biochem Biophys Res Commun. 2010;403(3–4):435–41. 10.1016/j.bbrc.2010.11.050 21094149

[26] Hirasawa M, Schurmann P, Jacquot JP, Manieri W, Jacquot P, Keryer E, et al Oxidation-reduction properties of chloroplast thioredoxins, ferredoxin:thioredoxin reductase, and thioredoxin f-regulated enzymes. Biochemistry. 1999;38(16):5200–5. 10.1021/bi982783v 10213627

[27] Krimm I, Lemaire S, Ruelland E, Miginiac-Maslow M, Jaquot JP, Hirasawa M, et al The single mutation Trp35—>Ala in the 35–40 redox site of *Chlamydomonas reinhardtii* thioredoxin h affects its biochemical activity and the pH dependence of C36-C39 1H-13C NMR. Eur J Biochem. 1998;255(1):185–95. 9692918 10.1046/j.1432-1327.1998.2550185.x

[28] Lyles MM, Gilbert HF. Catalysis of the oxidative folding of ribonuclease A by protein disulfide isomerase: pre-steady-state kinetics and the utilization of the oxidizing equivalents of the isomerase. Biochemistry. 1991;30(3):619–25. 1988051 10.1021/bi00217a005

[29] Couturier J, Koh CS, Zaffagnini M, Winger AM, Gualberto JM, Corbier C, et al Structure-function relationship of the chloroplastic glutaredoxin S12 with an atypical WCSYS active site. J Biol Chem. 2009;284(14):9299–310. 10.1074/jbc.M807998200 19158074 PMC2666582

[30] Navrot N, Collin V, Gualberto J, Gelhaye E, Hirasawa M, Rey P, et al Plant glutathione peroxidases are functional peroxiredoxins distributed in several subcellular compartments and regulated during biotic and abiotic stresses. Plant Physiol. 2006;142(4):1364–79. 10.1104/pp.106.089458 17071643 PMC1676047

[31] Issakidis E, Lemaire M, Decottignies P, Jacquot JP, Miginiac-Maslow M. Direct evidence for the different roles of the N- and C-terminal regulatory disulfides of sorghum leaf NADP-malate dehydrogenase in its activation by reduced thioredoxin. FEBS Lett. 1996;392(2):121–4. 8772188 10.1016/0014-5793(96)00801-0

[32] Lappi AK, Lensink MF, Alanen HI, Salo KE, Lobell M, Juffer AH, et al A conserved arginine plays a role in the catalytic cycle of the protein disulphide isomerases. J Mol Biol. 2004;335(1):283–95. 14659757 10.1016/j.jmb.2003.10.051

[33] Dyson HJ, Jeng MF, Tennant LL, Slaby I, Lindell M, Cui DS, et al Effects of buried charged groups on cysteine thiol ionization and reactivity in *Escherichia coli* thioredoxin: structural and functional characterization of mutants of Asp 26 and Lys 57. Biochemistry. 1997;36(9):2622–36. 10.1021/bi961801a 9054569

[34] Chibani K, Tarrago L, Gualberto JM, Wingsle G, Rey P, Jacquot JP, et al Atypical thioredoxins in poplar: the glutathione-dependent thioredoxin-like 2.1 supports the activity of target enzymes possessing a single redox active cysteine. Plant Physiol. 2012;159(2):592–605. 10.1104/pp.112.197723 22523226 PMC3375927

[35] Ren G, Stephan D, Xu Z, Zheng Y, Tang D, Harrison RS, et al Properties of the thioredoxin fold superfamily are modulated by a single amino acid residue. J Biol Chem. 2009;284(15):10150–9. 10.1074/jbc.M809509200 19181668 PMC2665069

[36] Hirasawa M, Ruelland E, Schepens I, Issakidis-Bourguet E, Miginiac-Maslow M, Knaff DB. Oxidation-reduction properties of the regulatory disulfides of sorghum chloroplast nicotinamide adenine dinucleotide phosphate-malate dehydrogenase. Biochemistry. 2000;39(12):3344–50. 10727227 10.1021/bi9916731

[37] Tavender TJ, Springate JJ, Bulleid NJ. Recycling of peroxiredoxin IV provides a novel pathway for disulphide formation in the endoplasmic reticulum. Embo J. 2010;29(24):4185–97. 10.1038/emboj.2010.273 21057456 PMC3018787

[38] Zhu L, Yang K, Wang X, Wang CC. A novel reaction of peroxiredoxin 4 towards substrates in oxidative protein folding. PLoS One. 2014;9(8):e105529 10.1371/journal.pone.0105529 25137134 PMC4138195

[39] Chambers JE, Tavender TJ, Oka OB, Warwood S, Knight D, Bulleid NJ. The reduction potential of the active site disulfides of human protein disulfide isomerase limits oxidation of the enzyme by Ero1alpha. J Biol Chem. 2010;285(38):29200–7. 10.1074/jbc.M110.156596 20657012 PMC2937950

[40] Onda Y, Kobori Y. Differential activity of rice protein disulfide isomerase family members for disulfide bond formation and reduction. FEBS Open Bio. 2014;4:730–4. 10.1016/j.fob.2014.07.007 25161881 PMC4141933

[41] Tian G, Xiang S, Noiva R, Lennarz WJ, Schindelin H. The crystal structure of yeast protein disulfide isomerase suggests cooperativity between its active sites. Cell. 2006;124(1):61–73. 10.1016/j.cell.2005.10.044 16413482

[42] Kozlov G, Maattanen P, Schrag JD, Pollock S, Cygler M, Nagar B, et al Crystal structure of the bb' domains of the protein disulfide isomerase ERp57. Structure. 2006;14(8):1331–9. 10.1016/j.str.2006.06.019 16905107

[43] Maattanen P, Kozlov G, Gehring K, Thomas DY. ERp57 and PDI: multifunctional protein disulfide isomerases with similar domain architectures but differing substrate-partner associations. Biochemistry and cell biology. 2006;84(6):881–9. 10.1139/o06-186 17215875

[44] Alanen HI, Williamson RA, Howard MJ, Lappi AK, Jantti HP, Rautio SM, et al Functional characterization of ERp18, a new endoplasmic reticulum-located thioredoxin superfamily member. J Biol Chem. 2003;278(31):28912–20. 10.1074/jbc.M304598200 12761212

[45] Hatahet F, Ruddock LW. Protein disulfide isomerase: a critical evaluation of its function in disulfide bond formation. Antioxid Redox Signal. 2009;11(11):2807–50. 10.1089/ARS.2009.2466 19476414

[46] Lafaye C, Iwema T, Carpentier P, Jullian-Binard C, Kroll JS, Collet JF, et al Biochemical and structural study of the homologues of the thiol-disulfide oxidoreductase DsbA in *Neisseria meningitidis*. J Mol Biol. 2009;392(4):952–66. 10.1016/j.jmb.2009.07.056 19631659

[47] Menchise V, Corbier C, Didierjean C, Saviano M, Benedetti E, Jacquot JP, et al Crystal structure of the wild-type and D30A mutant thioredoxin h of *Chlamydomonas reinhardtii* and implications for the catalytic mechanism. Biochem J. 2001;359(Pt 1):65–75. 11563970 10.1042/0264-6021:3590065 PMC1222122

[48] Bisio H, Bonilla M, Manta B, Grana M, Salzman V, Aguilar PS, et al A New Class of Thioredoxin-Related Protein Able to Bind Iron-Sulfur Clusters. Antioxid Redox Signal. 2015. 10.1089/ars.2015.6377 PMC6913166 26381228

[49] Couturier J, Stroher E, Albetel AN, Roret T, Muthuramalingam M, Tarrago L, et al Arabidopsis chloroplastic glutaredoxin C5 as a model to explore molecular determinants for iron-sulfur cluster binding into glutaredoxins. J Biol Chem. 2011;286(31):27515–27. 10.1074/jbc.M111.228726 21632542 PMC3149344

[50] Mesecke N, Mittler S, Eckers E, Herrmann JM, Deponte M. Two novel monothiol glutaredoxins from Saccharomyces cerevisiae provide further insight into iron-sulfur cluster binding, oligomerization, and enzymatic activity of glutaredoxins. Biochemistry. 2008;47(5):1452–63. 10.1021/bi7017865 18171082

[51] Rouhier N, Unno H, Bandyopadhyay S, Masip L, Kim SK, Hirasawa M, et al Functional, structural, and spectroscopic characterization of a glutathione-ligated [2Fe-2S] cluster in poplar glutaredoxin C1. Proc Natl Acad Sci U S A. 2007;104(18):7379–84. 10.1073/pnas.0702268104 17460036 PMC1863468

[52] Couturier J, Touraine B, Briat JF, Gaymard F, Rouhier N. The iron-sulfur cluster assembly machineries in plants: current knowledge and open questions. Front Plant Sci. 2013;4:259 10.3389/fpls.2013.00259 23898337 PMC3721309

[53] Lundström-Ljung J, Birnbach U, Rupp K, Soling HD, Holmgren A. Two resident ER-proteins, CaBP1 and CaBP2, with thioredoxin domains, are substrates for thioredoxin reductase: comparison with protein disulfide isomerase. FEBS Lett. 1995;357(3):305–8. 7835433 10.1016/0014-5793(94)01386-f

[54] Jessop CE, Watkins RH, Simmons JJ, Tasab M, Bulleid NJ. Protein disulphide isomerase family members show distinct substrate specificity: P5 is targeted to BiP client proteins. J Cell Sci. 2009;122(Pt 23):4287–95. 10.1242/jcs.059154 19887585 PMC2779130

[55] Appenzeller-Herzog C, Ellgaard L. In vivo reduction-oxidation state of protein disulfide isomerase: the two active sites independently occur in the reduced and oxidized forms. Antioxid Redox Signal. 2008;10(1):55–64. 10.1089/ars.2007.1837 17939758

[56] Schulman S, Wang B, Li W, Rapoport TA. Vitamin K epoxide reductase prefers ER membrane-anchored thioredoxin-like redox partners. Proc Natl Acad Sci U S A. 2010;107(34):15027–32. 10.1073/pnas.1009972107 20696932 PMC2930587

[57] Tavender TJ, Sheppard AM, Bulleid NJ. Peroxiredoxin IV is an endoplasmic reticulum-localized enzyme forming oligomeric complexes in human cells. Biochem J. 2008;411(1):191–9. 10.1042/BJ20071428 18052930 PMC4864507

[58] Wajih N, Hutson SM, Wallin R. Disulfide-dependent protein folding is linked to operation of the vitamin K cycle in the endoplasmic reticulum. A protein disulfide isomerase-VKORC1 redox enzyme complex appears to be responsible for vitamin K1 2,3-epoxide reduction. J Biol Chem. 2007;282(4):2626–35. 10.1074/jbc.M608954200 17124179

[59] Gross E, Kastner DB, Kaiser CA, Fass D. Structure of Ero1p, source of disulfide bonds for oxidative protein folding in the cell. Cell. 2004;117(5):601–10. 15163408 10.1016/s0092-8674(04)00418-0

[60] Kikuchi M, Doi E, Tsujimoto I, Horibe T, Tsujimoto Y. Functional analysis of human P5, a protein disulfide isomerase homologue. J Biochem. 2002;132(3):451–5. 12204115 10.1093/oxfordjournals.jbchem.a003242

[61] Lennon BW, Williams CH Jr., Ludwig ML. Twists in catalysis: alternating conformations of *Escherichia coli* thioredoxin reductase. Science. 2000;289(5482):1190–4. 10947986 10.1126/science.289.5482.1190

